# AGREE II for TCM: Tailored to evaluate methodological quality of TCM clinical practice guidelines

**DOI:** 10.3389/fphar.2022.1057920

**Published:** 2023-01-12

**Authors:** Xiuli Xie, Yangyang Wang, Hui Li

**Affiliations:** ^1^ Department of Standardization of Chinese Medicine, The Second Affiliated Hospital of Guangzhou University of Chinese Medicine, Guangdong Provincial Hospital of Chinese Medicine, Guangzhou, China; ^2^ Department of Standardization of Chinese Medicine, Guangdong Provincial Academy of Chinese Medicine, Guangzhou, China; ^3^ State Key Laboratory of Dampness Syndrome of Chinese Medicine, The Second Affiliated Hospital of Guangzhou University of Chinese Medicine, Guangzhou, China

**Keywords:** agree Ⅱ, adjust and expand, guidelines, quality, evaluation

## Abstract

**Background:** The *Appraisal of Guidelines Research and Evaluation* (AGREE) II instrument has been widely used in the methodological quality assessment of clinical practice guidelines (CPG). Chinese medicine CPGs have unique characteristics which distinguish them from those of Western medicine, e.g. syndrome differentiation, on which treatments are based. As such, certain domains and items in AGREE II are unsuitable for assessing TCM CPGs. Therefore, it is necessary to adjust and supplement the description and rating section of some items of the AGREE Ⅱinstrument.

**Purpose:** To adjust and expand AGREE II according to characteristics of TCM clinical practice guidelines.

**Methods:** A research working group was established, consisting of a core working group and an expert consensus group, before a systematic literature search performed to screen for TCM guidelines. Two researchers evaluated the quality of the included guidelines using AGREE Ⅱ and later proposed adjustments to some items of AGREE Ⅱ and supplementary comments, which were applicable to TCM CPGs, and drafted an initial version of AGREE Ⅱ for TCM. Suggestions from literature on development and evaluation of TCM CPGs were solicited and integrated into the revised version, which 16 experts were then invited to advise on. When the experts reached a consensus, their comments to the draft were adopted by the core group into the final version.

**Results:** After evaluating the included TCM guidelines, the two researchers offered adjustments and supplementary comments for AGREE Ⅱ Items 1, 7, 10, 11, 12, 15, and 18, and drafted an initial version of AGREE Ⅱ for TCM. Combining suggestions from the literature on development and quality evaluation of TCM clinical guidelines, the core working group modified AGREE Ⅱ items 2, 4, 5, 8, 9, 13, 20, and 21, then proposed the revised version of AGREE Ⅱ for TCM, on which was advised by a group of experts, before consensus on improvements was reached. The results of the first round of expert surveys showed strong agreement, and experts’ opinions were adopted into the final version of AGREE Ⅱ for TCM.

**Conclusion:** Based on the characteristics of the TCM CPGs, we adjustment and expansion were made to create AGREE II for TCM. This version is suitable for the assessment of methodological quality of TCM CPGs, capable of providing content support for the standardization of procedures and methods of formulating TCM CPGs.

## 1 Introduction

Clinical practice guidelines (CPGs) are recommendations for providing patients with the optimal healthcare services (Institute of Medicine, 2011). They are the core technical standard in the field of medicine. Traditional Chinese medicine (TCM) and Western medicine have long complemented each other in promoting the health of the people in China, which has become a significant advantage of “health services with Chinese characteristics”. In recent years, with the Central government’s promotion of TCM, there has been a proliferation of CPGs for TCM, of which, however, the quality varies. Moreover, the development of some guidelines lacked scientific normativity (Huang et al., 2018). Studies pointed out that there remains a wide gap between the methodological quality of TCM clinical guidelines and the international ones. Problems in quality existing requires effective measures (Yao, 2016). Additionally, rate of application for clinical guidelines is not promising, and some guidelines do not serve as a guidance for clinical practice (Wu et al., 2016).

Appropriate methodology and strict formulation strategy are not only crucial for drafting quality guidelines, but also conducive for implementing the guidelines’ recommendations within (Burgers et al., 2004). Guideline evaluation is an effective way to improve guideline quality and provide necessary reference for clinical application. Many researchers believe that scientifically valid methods for rational evaluation of guidelines are needed so that recommendations can be made for revising or updating guideline (Jiang and Chen 2016; Li et al., 2016; Huang et al., 2018). The *Appraisal of Guidelines for Research and Evaluation* instrument, second edition (AGREE Ⅱ) (The AGREE Next Steps Consortiu, 2009) is recognized as the international “gold standard” of guideline evaluation (Brouwers et al., 2012). At present, it is widely used in the field of guideline development and evaluation.

Unlike current medical guidelines, TCM guidelines have distinctive characteristics, including, for example, treatment based on syndrome differentiation, Chinese Medicine formulas and traditional therapy, *etc.* The prescription of medication in TCM is based on the results of syndrome differentiation. There is a clear corresponding relationship between the etiology and pathogenesis, syndrome differentiation and treatment formulas. This direct cause-and-effect relationship should be presented in the TCM guidelines. The content of ancient texts and the experience of famous TCM practitioners are often essential in the formation of TCM guidelines. The above contents specific to TCM are not focused on in the AGREE II instrument. Therefore, we believe it is necessary to adjust and supplement the description and rating section of some items of the AGREE Ⅱinstrument to be applicable to the evaluation of TCM guidelines. Study showed that existing domestic and international guideline evaluation instruments (including AGREE II) are not directly applicable to TCM guidelines due to certain limitations. For example, a 7-point scale is used in some instruments, but scoring criteria for each value is not described; some evaluation fields do not conform to conditions in China; some items do not apply to the field of TCM (Bai et al., 2020a). At present, there is no relevant research on whether AGREE II is applicable to TCM CPGs. In only one study, AGREE II was used to evaluate the methodological quality of TCM CPGs, but there was no further research on its applicability to evaluating TCM CPGs (Yao, 2016). A guideline quality evaluation system in line with the characteristics of TCM may not only guide the process and methods of formulating guidelines regarding TCM diagnosis and treatment, but also promote scientific development of methodology of formulating TCM clinical practices. In this study, we attempted to adjust and expand the description and rating section of some items of the AGREE Ⅱinstrument to cover the methodological quality assessment of TCM CPGs based on their characteristics, which may promote the accurate assessment of TCM CPGs, and the presentation of opinions that help improve the procedures and methods for TCM CPGs.

## 2 Methods

### 2.1 Establishing a research working group

Firstly, we established a research working group for this study. It consisted of: 1) A core working group, including professionals in guideline methodology, evidence-based medicine, and clinicians, who were responsible for searching literature, establishing initial items, and collecting and statistically analyzing questionnaires, and writing reports. Each member was required to have experience using AGREE II to evaluate guidelines; 2) An expert consensus group, including guideline methodology experts, evidence-based medicine experts, and clinical experts, who were responsible for evaluating and screening the evaluation instrument’s items.

### 2.2 Guideline evaluation

#### 2.2.1 Systematic search for guidelines

On 26 March 2020, we performed a systematic literature search from inception till now with the keywords “Guideline*“, “recommendation*“, “statement”, “regulation”, “consensus”, “Chinese medicine”, “Chinese herb” and “TCM”. We searched seven computerized databases [Embase, PubMed and Web of Science, CBM (Chinese Biomedical Literature Database), CNKI (China National Knowledge Infrastructure) and Wanfang Data]; four guideline databases [NGC(National Guideline Clearinghouse), GIN (Guidelines International Network) and NICE (National Institute for Health and Care Excellence)]; one standard database (National public service platform for standard information); and four online book malls.

#### 2.2.2 Guideline screening

The inclusion criteria are as follows: 1) Literature that meets the definition of guideline in AGREE II (CPGs were systematically developed statements to assist practitioner and patient decisions about appropriate healthcare for specific clinical circumstances) (Woolf et al., 1999); 2) Article titles containing “TCM guideline”, or the names of publishing organizations containing “Chinese Medicine”; 3) Issued by official organizations. Exclusion criteria include: 1) abstracts; 2) bibliographic guidelines; 3) outdated, translated or adapted versions of guidelines; 4) acupuncture guidelines. We classified all retrieved guidelines by publishing organizations. In the first round of guideline screening, one guideline from each organization on different specialties would be selected, to ensure that the specialties of each guide were as different as much as possible. Then we analyzed if any specialties were missed. In the second round, we screened guidelines, issued by organizations having published more than two guidelines, for ones that focused on specialties left out in the first round. For the guidelines published by the same institution that are combined into books, only one of the guidelines for a particular specialty are selected.

#### 2.2.3 Evaluation of guideline quality

Two trained reviewers from the core working group (Xiuli Xie, Yangyang Wang) who were familiar with guideline development methodology independently apply AGREE Ⅱ to evaluate the eligible CPGs’ methodological quality. The reviewers analyzed which TCM guideline characteristics were different from modern medical guidelines. Finally, based on the evaluation of the TCM guidelines experience, the two reviewers suggested adjustments and supplementary comments to some items of AGREE Ⅱ, and drafted an initial version of AGREE Ⅱ for TCM.

### 2.3 Systematic search for literature on TCM clinical guideline development and evaluation

#### 2.3.1 Literature retrieval

On 21 May 2020, we performed a systematic literature search with the keywords “Guideline*“, “Chinese medicine”, “Chinese herb”, “TCM”, “development”, “evaluation” and “methodology”. The search date range began with each database’s inception date. We used four Chinese computerized databases: CBM, CNKI and Wanfang Data.

#### 2.3.2 Literature screening

The inclusion criteria are literature on the development and evaluation of TCM clinical guidelines, including but not limited to the literature about evaluation by AGREE II. A total of 40 articles were included. We extracted the results of TCM clinical guideline quality evaluations in all the literature to establish a database, giving special attention to any material on deficiencies in TCM clinical guidelines and difficulties about the quality of TCM clinical guidelines. We also refer to the difficulties and solutions of establishing quality evaluation instruments (Bai et al., 2020b), applicability evaluation instruments (Bai et al., 2020c) and guideline reporting standards (Xia, 2019) for TCM guidelines in the study on how to make the evaluation instruments and reporting standards conform to TCM’s characteristics. All material in the database was classified according to the AGREE Ⅱ items.

The members of the core working group held a meeting to discuss the initial version of AGREE Ⅱ for TCM Further suggestions were proposed for adjustments and additions to improve the initial version, which were adopted into a revised version.

### 2.4 Expert consensus

Expert consensus method was applied, consisting of one or two rounds of expert investigation in the form of electronic questionnaire. The composition of the expert group is shown in Supplementary File S3. If experts’ opinions were convergent after the first round of the survey, the second round of investigation would be skipped. We asked experts to score the importance of each item. For the evaluation index, we adopted Likert’s five scale scoring method (Dawes, 2008), which were “strongly agree” “agree”, “somewhat agree”, “disagree” and “strongly disagree”, assigned 5, 4, 3, two and one points, respectively.

If not agreeing to certain content of the items completely, they would be invited to offer advice to modify it. Next, questionnaires were collected and sorted. The team members would then optimize the evaluation items’ content according to the results and drafted the final version of AGREE Ⅱ for TCM based on the expert consensus reached.

### 2.5 Statistical analysis

We analyzed expert consensus using the degree of expert positivity and authority (Zeng, 1996)**.** Expert positivity coefficient was the recovery rate for effective questionnaires. The principles of judging a questionnaire invalid were as follows: 1) Unqualified answers accounted for a large proportion of the questionnaire. For example, if all evaluated items in a questionnaire received full points, it would be judged invalid. 2) The percentage of missing answers to key variables in a questionnaire exceeded 15%.

Experts’ degree of authority is expressed by the expert authority coefficient (Cr), which is generally determined by two factors: one is the basis for experts to make judgments on problems, as represented by Ca, and the other is the coefficient of experts’ familiarity with indicators, represented by Cs. Authority equals to half of the sum of judgment coefficient and familiarity, that is:

$$Cr=\left(Ca+Cs\right)/2$$

The authority of experts is primarily based on self-evaluation. There is a functional relationship between experts’ authority and the prediction accuracy, with the latter increases along with the former. Furthermore, evaluation basis involves four dimensions: theoretical analysis, practical experience, knowledge from peers, and intuition. Each dimension is divided into three levels: high, medium and low, according to the degree of influence on expert judgment. The values are as follows (Zeng, 1996): theoretical analysis (0.3, 0.2, 0.1), practical experience (0.5, 0.4, 0.3), knowledge from peers (0.1, 0.1, 0.1) and intuition (0.1, 0.1, 0.1, 0.1). The degree of familiarity can be divided into five grades: 1, 0.8, 0.6, 0.4, and 0.2.

Statistical analysis was conducted in Statistical Package for the Social Sciences (SPSS) 17.0. Concentration degree for experts’ opinions was represented by arithmetic mean, grade sum and full score rate; coordination degree for experts’ opinions was represented by a variation coefficient and a coordination coefficient.

## 3 Results

### 3.1 Establishing the initial version

A total of 518 sets of guidelines were included in this study, of which 17 sets were published in English and 501 were published in Chinese. They were developed by 19 organizations. After analysis, similarity was found among guidelines issued by a same organization, in terms of their development method, reporting structure and form. Therefore, it was unnecessary to include guidelines issued by a same organization,, while the comprehensiveness of the clinical specialties involved, and year of publication involved should be taken into consideration. We selected guidelines published in the recent 10 years whenever possible. Eleven organizations published only one guideline, while some published more than two, with the organization that published the most TCM guidelines being China association of Chinese medicine, having issued 465 guidelines. Numbers of guidelines developed by different organizations are shown in Supplementary File S1. After two rounds of screening, we eventually included a total of 26 sets of guidelines for evaluation (Chen et al., 2017; Sun et al., 2014; Linda et al., 2017; Geng et al., 2019; Fang and Wang, 2018; Liu et al., 2014; Li et al., 2015; Mao and Zhu, 2014; Fang et al., 2017; Wang and Xue, 2015; Li 2016; Chen et al., 2015; Li and Zhang, 2020; Yao et al., 2018; Li and Wang, 2016; Lin et al., 2019; Integrative Medicine Group of the Eighth Committee of the Chinese Medical Association, Family Planning Branch, 2019; China association of Chinese Medicine, 2011a; China association of Chinese Medicine, 2011b; Key Research Unit of COPD Lung-Qi Deficiency Syndrome et al., 2015; Guangdong Bureau of Quality and Technical Supervision, 2014; Guangdong Provincial Association of Chinese Medicine, 2021; Jiaxing Standard Quality Construction Promotion Association, 2021; Chinese Medicine and Bone Disease Discipline Group et al., 2015; Guo et al., 2014; China Association of Chinese Medicine, 2018) (Supplementary File S2). Based on the evaluation of the TCM guideline experience, the two evaluators offered adjustments and supplementary comments which were applicable to evaluating TCM CPGs for the “user’s manual description” section and “how to rate” section of some items of the AGREE Ⅱ instrument. The following items are included: Domain 1 (Item 1), Domain 3 (Items 7, 10, 11, 12), Domain 4 (Item 15), and Domain 5 (Item 18) (Table 1). We then drafted the initial version of AGREE Ⅱ for TCM.

**TABLE 1:** The final version of AGREE Ⅱ for TCM Instrument.

Item	Descriptions and scoring criteria
**Domain 1. Scope and purpose**	
1	**The overall objective(s) of the guidelines are clarified.**
	**Description:**
	This addresses the potential health effects of a set of guidelines on individuals, patient populations, and society as a whole. The overall objective(s) of the guidelines must be described in detail. For example, guideline contents include disease diagnosis, TCM syndrome diagnosis, treatment and prevention. The expected health benefits from the guidelines must be specific to the clinical problem or health topic. If it is a set of TCM guidelines for a certain disease, the purpose and advantages of TCM treatment must be clarified; the guidelines for using the disease name in Western medicine must specify the corresponding TCM disease name, and the guidelines for using the traditional Chinese medicine disease name must also specify the corresponding disease scope in Western medicine, For example, specific statements would be:
	• These guidelines are applicable to patients with psoriasis vulgaris, which is known by names such as psoriasis vulgaris, tinea pinealis, dry tinea and snake lice.
	• These guidelines are applicable to cough, and common in Western medicine, such as in colds, acute bronchitis, chronic bronchitis, cough variant asthma and postnasal drip syndrome.
	• In treating this disease, TCM has a clear curative effect, can reduce skin lesions from erythema, scales and itching symptoms, reduce the disease recurrence, and delay the spread of the disease to other parts of the body.
	**Rating:**
	Item content includes the following criteria:
	• health intent(s) (i.e., prevention, screening, diagnosis, treatment, prevention, etc.)
	• The disease names in both traditional Chinese medicine and Western medicine must be clarified in the guidelines (if appropriate).
	• advantages, expected benefits or outcomes of TCM treatment
	• target(s) (e.g., patient population, society)
	Scoring:7 points will be awarded if all the content listed in “Evaluation” is explained in detail. 2-3 points will be deducted if information related to a criterium under an Item is incomplete. Points will be deducted cumulatively according to how many criteria are not met, and only 1 point will be awarded if no content is described.
2	The health question(s) covered by the guidelines are clarified.
	**Description:**
	A detailed description of the health questions covered by the guidelines must be provided, particularly for the key recommendations (see Item 17). For example, specific statements would be:
	• Can Chinese native medicine wash-outside in psoriasis treatment reduce the disease’s frequency?
	• Can biological agents combined with Chinese herbal decoction flakes improve the curative effect in plaque psoriasis treatment?
	**Rating:**
	Clinical problems cause PICO problems. Item content includes the following criteria:
	• Target population (P)
	• intervention(s) or exposure(s) (e.g., Chinese herbal decoction flakes, Chinese patent
	• medicine or
	• Chinese medicine injection) (I)
	• comparisons (if appropriate) (C)
	• outcome(s) (Important patient outcome indicators should reflect the advantages of TCM treatment) (O)
	• healthcare setting or context
	Scoring: 7 points will be awarded if all the content listed in “Evaluation” is explained in detail. 2-3 points will be deducted if information related to a criterium under an Item is incomplete. Points will be deducted cumulatively according to how many criteria are not met, and only 1 point will be awarded if no content is described.
3	**The population (patients, the general public, etc.) to whom the guidelines are meant to apply is described.**
	**Description:** No change^a^
	**Rating:**
	The item’s content must include the following criteria: No change
	Scoring:7 7 points will be awarded if all the content listed in “Evaluation” is explained in detail. 2-3 points will be deducted if information related to a criterium under an Item is incomplete. Points will be deducted cumulatively according to how many criteria are not met, and only 1 point will be awarded if no content is described.
**Domain 2. Stakeholder involvement**	
4	**The guideline development group includes individuals from all relevant professional groups.**
	**Description:**
	This item refers to the professionals who were involved at some stage of the development process. This may include members of the steering committee, the research team involved in selecting and reviewing/rating the evidence, and individuals involved in formulating the final recommendations. This item excludes individuals who have externally reviewed the guidelines (see Item 13). It also excludes target population representation (see Item 5). Information about the guideline development group’s composition, discipline, and relevant expertise also must be provided.
	**Rating:**
	The item’s content must include the following criteria:
	1) For each member of the guideline development group, the following information is included:
	• name
	• discipline/content expertise (e.g., neurosurgeon, methodologist)
	• institution (e.g., St. Peter’s Hospital)
	• geographical location (e.g., Seattle, WA)
	• a description of the member’s role in the guideline development group
	2) Are the members an appropriate match for the topic and scope? The members of the guideline development group must include experts of TCM or integrated traditional Chinese and Western medicine and experts of Chinese herbs. Other potential candidates include relevant clinicians and nurses, content experts, researchers, policy makers, clinical administrators.
	3) Is there at least one methodology expert included in the development group (e.g., systematic review expert, epidemiologist, statistician, library scientist, etc.)?
	Scoring: 7 points will be awarded if all the content listed in “Evaluation” is explained in detail. 2-3 points will be deducted if information related to a criterium under an Item is incomplete. Points will be deducted cumulatively according to how many criteria are not met, and only 1 point will be awarded if no content is described.
5	**The views and preferences of the target population (patients, the general public, etc.) have been sought.**
	**Description:**
	Information about the target population’s healthcare experiences and expectations should inform guideline development. There are various methods for ensuring that these perspectives inform the different stages of guideline development for stakeholders. Examples would include formal consultations with patients/the general public to determine priority topics, these stakeholders’ participation in the guideline development group, or external review by these stakeholders on draft documents. Alternatively, information could be obtained from interviews of these stakeholders, or from literature reviews of patient/general public’s values, preferences or experiences. There must be evidence that some process has taken place, and that stakeholders’ views have been considered.
	**Rating:**
	• Item content must include the following criteria:
	• The target population for collecting information must have experience with TCM diagnosis.
	• statement of strategy used to capture patients’/the general public’s views and preferences (e.g., participation in the guideline development group, literature review of values and preferences, or questionnaire survey)
	• outcomes/information gathered from patient/public information
	• description of how the information gathered informed the guideline development process, and/or
	• drafting the recommendations
	Scoring: 7 points will be awarded if all the content listed in “Evaluation” is explained in detail. 2-3 points will be deducted if information related to a criterium under an Item is incomplete. Points will be deducted cumulatively according to how many criteria are not met, and only 1 point will be awarded if no content is described.
6	**The guidelines’ target users are clearly defined.**
	**Description:**
	The target users must be clearly defined in the guidelines, so that the reader can immediately determine if the guidelines are relevant to them. For example, *TCM Guidelines for Diagnosis and Treatment of Common Diseases in Internal Medicine – Headaches* is applicable to the clinical diagnosis and treatment of headaches in TCM. It is applicable to all levels of TCM (Integrated Traditional Chinese and Western Medicine) medical institutions, as well as medical institutions offering TCM services. These guidelines’ target users may include Chinese medicine practitioners (Integrated Traditional Chinese and Western Medicine) and licensed assistant Chinese medicine practitioners (with the exception of Pediatrics). Clinical practitioners can also refer to them.
	**Rating:**
	The item’s content must include the following criteria:
	• a clear description of the intended guideline audience (e.g. specialists, family physicians, patients, clinical or institutional leaders/administrators)
	• a description of how the guidelines may be used by their target audience (e.g., to inform clinical decisions, to inform policy, to inform standards of care)
	Scoring: 7 points will be awarded if all the content listed in “Evaluation” is explained in detail. 2-3 points will be deducted if information related to a criterium under an Item is incomplete. Points will be deducted cumulatively according to how many criteria are not met, and only 1 point will be awarded if no content is described.
**Domain 3. Rigor of development**	
7	**Systematic methods were used to search for evidence.**
	**Description**
	Details of the strategy used to search for evidence must be provided; including the search terms used, sources consulted, and dates of the literature covered. Sources may include the tracing of ancient Chinese medical literature records and the searching for modern medical literature. Sources include ancient Chinese medical literature electronic databases (e.g., Chinese Medical Dictionary), Chinese electronic scholarly databases (e.g., CMKI, Wanfang, Wipu, CBM), English electronic scholarly databases (e.g., MEDLINE, ENBASE, CINAHL), other sources including systematic review databases (e.g., Cochrane Library, DARE), manually searched journals, conference proceedings, and other guidelines (e.g., the US National Guideline Clearinghouse, the German Guidelines Clearinghouse). The search terms must include the Chinese Medicine disease name and search terms such as "TCM, Chinese medicine, proprietary Chinese medicine, herbal medicine". The search strategy should be as comprehensive as possible and executed in a manner free from potential biases and detailed enough to be replicable.
	**Rating**
	The item’s content must include the following criteria:
	• named electronic database(s) or evidence source(s) where the search was performed, ancient Chinese medical literature electronic databases (e.g., Chinese Medical Dictionary), Chinese electronic databases (e.g., CMKI, Wanfang, Wipu, CBM), English electronic databases (e.g., MEDLINE, ENBASE, CINAHL).
	• time periods searched (e.g., January 1, 2004 to March 31, 2008)
	• search terms used (e.g., text words, indexing terms, sub-headings)
	• full search strategy (e.g., possibly located in the appendix)
	Scoring: 7 points will be awarded if all the content listed in “Evaluation” is explained in detail. 2-3 points will be deducted if information related to a criterium under an Item is incomplete. Points will be deducted cumulatively according to how many criteria are not met, and only 1 point will be awarded if no content is described.
8	**The criteria for selecting the evidence are clarified.**
	**Description**
	Criteria for the inclusion and exclusion of evidence at the time of search must be provided. These criteria and the reasons for exclusion/inclusion of evidence must be clarified. For example, the authors of a set of guidelines may decide to include only evidence from randomized controlled trials, and exclude non-Chinese literature.
	**Rating**
	The item’s content must include the following criteria:
	• description of the inclusion criteria, including:
	➢ target population (patient, the general public, etc.) characteristics
	➢ study design
	➢ comparisons (if relevant)
	➢ outcomes
	➢ language (if relevant)
	➢ context (if relevant)
	• description of the exclusion criteria (if relevant; e.g., Chinese-language-only listed in the inclusion criteria statement could logically preclude non-Chinese-language from being listed in the exclusion criteria statement)
	Scoring: 7 points will be awarded if all the content listed in “Evaluation” is explained in detail. 2-3 points will be deducted if information related to a criterium under an Item is incomplete. Points will be deducted cumulatively according to how many criteria are not met, and only 1 point will be awarded if no content is described.
9	**The strengths and limitations of the body of evidence are clarified.**
	Description: No change
	**Rating:** No change
	Scoring: 7 points will be awarded if all the content listed in “Evaluation” is explained in detail. 2-3 points will be deducted if information related to a criterium under an Item is incomplete. Points will be deducted cumulatively according to how many criteria are not met, and only 1 point will be awarded if no content is described.
10	**The methods for formulating the recommendations are clarified.**
	**Description**
	A description of the methods used to formulate the recommendations and how final decisions were reached should be provided. For example, methods may include a voting system, informal consensus, and formal consensus techniques (e.g., Delphi, Glaser techniques). Experts participating in the drafting of recommendations must include experts of TCM, integrated traditional Chinese and Western medicine. Any areas of disagreement and the methods of resolving them should be specified. The TCM diagnosis must have clear sources and basis, such as expert consensus or literature.
	**Rating**
	The item’s content must include the following criteria:
	• description of the recommendation development process (e.g., background information on the experts who participate in drafting the recommendations; the members of the expert group must include experts in TCM or integrative Chinese and Western medicine; steps used in the modified Delphi technique; voting procedures that were considered)
	• outcomes of the recommendation development process (e.g., the extent to which consensus was reached using the modified Delphi technique, outcome of voting procedures)
	• description of how the process influenced the recommendations (e.g., how the results of the Delphi technique influenced final recommendation, alignment with recommendations and the final vote)
	Scoring: 7 points will be awarded if all the content listed in “Evaluation” is explained in detail. 2-3 points will be deducted if information related to a criterium under an Item is incomplete. Points will be deducted cumulatively according to how many criteria are not met, and only 1 point will be awarded if no content is described.
11	**The health benefits, side effects, and risks have been considered in formulating the recommendations.**
	**Description**
	The guidelines should consider health benefits, side effects, and risks when formulating the recommendations.
	For example, a set of guidelines on breast cancer management may include a discussion on the overall effects on various final outcomes. These may include: survival, quality of life, adverse effects, and symptom management, or a discussion comparing one treatment option to another. There should be evidence that these issues have been addressed. The characteristics and advantages of traditional Chinese medicine treatment must be explained; the health benefits and deficiencies of any Chinese medicinal herb with toxic side effects must be analyzed.
	**Rating**
	The item’s content must include the following criteria:
	• supporting data and report of benefits
	• the characteristics and advantages of traditional Chinese medicine treatment
	• supporting data and report of harm/side effects/risks
	• reporting of the balance/trade-off between benefits and harm/side effects/risks, analysis of the health benefits and deficiencies of any Chinese medicinal herb with toxic side effects.
	• recommendations reflecting considerations of both benefits and harm/side effects/risks
	Scoring: 7 points will be awarded if all the content listed in “Evaluation” is explained in detail. 2-3 points will be deducted if information related to a criterium under an Item is incomplete. Points will be deducted cumulatively according to how many criteria are not met, and only 1 point will be awarded if no content is described.
12	**There is an explicit link between the recommendations and the supporting evidence.**
	**Description**
	An explicit link between the recommendations and the evidence on which they are based should be included in the guidelines. The user of the guidelines should be able to identify the components of the body of evidence relevant to each recommendation. There must be clear correspondence between the etiology and pathogenesis, syndrome differentiation and treatment principles, therapeutic formulas or proprietary Chinese medicine in TCM guidelines. The composition of the therapeutic formulas in the recommendations must have the same name and composition as those in the evidence.
	**Ratings**
	The item’s content must include the following criteria:
	• the guidelines describe how the guideline development group linked and used the evidence to inform recommendations (When evidence is lacking or a recommendation is informed primarily by consensus of opinion by the guideline group, rather than the evidence, is this clearly stated and described?)
	• each recommendation is linked to a key evidence description/paragraph and/or reference list
	• recommendations linked to evidence summaries, evidence tables in the results sections
	• of guidelines; the etiology and pathogenesis, syndrome differentiation and classification in the TCM guidelines have clear correspondence with treatment principles, treatment prescriptions or proprietary Chinese medicines
	• the composition of the therapeutic formula in the recommendation must have the same name and composition as the formula in the supporting evidence.
	Scoring: 7 points will be awarded if all the content listed in “Evaluation” is explained in detail. 2-3 points will be deducted if information related to a criterium under an Item is incomplete. Points will be deducted cumulatively according to how many criteria are not met, and only 1 point will be awarded if no content is described.
13	**The guidelines have been externally reviewed by experts prior to their publication.**
	**Description**
	Guidelines should be reviewed externally before they are published. Reviewers should not have been involved in the guideline development group. Reviewers should include experts in clinical TCM, as well as methodological experts. Target population representatives (e.g., patients, the general public) may also be included. A description of the methodology used to conduct the external review should be presented, which may include a list of the reviewers and their affiliations.
	**Rating**
	The item’s content must include the following criteria:
	• a clear description of the intended guideline audience’s (e.g., specialists, family physicians, patients, clinical or institutional leaders/administrators) purpose and intent regarding the external review (e.g., to improve quality, gather feedback on draft recommendations, assess applicability and feasibility, disseminate evidence)
	• methods utilized for the external review (e.g., rating scale, open-ended questions)
	• description of the external reviewers (e.g., number, type of reviewers, affiliations)
	• outcomes/information gathered from the external review (e.g., summary of key findings)
	• description of how the information gathered was used to inform the guideline development process and/or drafting of recommendations (e.g., the guideline panel considered the review results in drafting the final recommendations)
	Scoring: 7 points will be awarded if all the content listed in “Evaluation” is explained in detail. 2-3 points will be deducted if information related to a criterium under an Item is incomplete. Points will be deducted cumulatively according to how many criteria are not met, and only 1 point will be awarded if no content is described.
14	**A procedure for updating the guidelines is provided.**
	**Description:** No change
	**Rating**
	The item’s content must include the following criteria: No changes
	Scoring: 7 points will be awarded if all the content listed in “Evaluation” is explained in detail. 2-3 points will be deducted if information related to a criterium under an Item is incomplete. Points will be deducted cumulatively according to how many criteria are not met, and only 1 point will be awarded if no content is described.
**Domain 4. Clarity of presentation**	
15	**The recommendations are specific and unambiguous.**
	**Description**
	A recommendation must provide a concrete and precise description of which option is appropriate in which situation and in what population group, as informed by the body of evidence. The usage and dosage of the Chinese medicinal herb must be specified in the recommendation; the diagnostic criteria of TCM syndromes must be clear; there must be indications and detailed administration methods for TCM therapies; Chinese herbal medicines must have clear sources if they are non-self-formulated; there must be explanations for the special TCM terms that affect the guidelines.
	It is important to note that in some instances, evidence is not always clear-cut and there may be uncertainty about the best care option(s). In this case, the uncertainty should be stated in the guidelines.
	**Rating:**
	• The item’s content must include the following criteria:
	• statement of the recommended action
	• identification of the intent or purpose of the recommended action (e.g., to improve quality of life, to mitigate side effects)
	• the content of the recommendations is clear and unambiguous
	• identification of the relevant population (e.g., patients, the general public)
	• caveats or qualifying statements, if relevant (e.g., patients or conditions for whom the recommendations would not apply)
	Scoring: 7 points will be awarded if all the content listed in “Evaluation” is explained in detail. 2-3 points will be deducted if information related to a criterium under an Item is incomplete. Points will be deducted cumulatively according to how many criteria are not met, and only 1 point will be awarded if no content is described.
16	**The options for management of the condition or health issue are clearly presented**
	**Description**
	A set of guidelines that targets the management of a disease must consider all possible options for screening, prevention, diagnosis or treatment of the condition it covers. These possible options must be clearly presented in the guidelines.
	For example, a recommendation on the management of depression may contain the following treatment alternatives:
	a. Chinese medicine treatment
	b. Acupuncture
	c. Acupoint application
	**Rating**
	The item’s content must include the following criteria:
	• a clear description of the intended guideline audience (e.g. specialists, family physicians, patients, clinical or institutional leaders/administrators), a description of options
	• description of the population or clinical situation most appropriate for each option
	Scoring: 7 points will be awarded if all the content listed in “Evaluation” is explained in detail. 2-3 points will be deducted if information related to a criterium under an Item is incomplete. Points will be deducted cumulatively according to how many criteria are not met, and only 1 point will be awarded if no content is described.
17	**Key recommendations are easily identifiable**
	**Description:** No changes
	**Rating**
	The item’s content must include the following criteria: No changes
	Scoring: 7 points will be awarded if all the content listed in “Evaluation” is explained in detail. 2-3 points will be deducted if information related to a criterium under an Item is incomplete. Points will be deducted cumulatively according to how many criteria are not met, and only 1 point will be awarded if no content is described.
**Domain 5. Applicability**	
18	**The guidelines describe facilitators and barriers to their application.**
	**Description**
	There may be existing facilitators and barriers that will influence the application of guideline recommendations. For example:
	i. A set of guidelines on stroke may require that care be coordinated through stroke units and stroke services. There may be a special funding mechanism in the region to enable the creation of stroke units.
	ii. A set of guidelines on diabetes in primary care may require that patients are seen and followed up in diabetic clinics. There may be an insufficient number of clinicians available in a region to enable the establishment of clinics.
	**Rating**
	The item’s content must include the following criteria:
	• identification of the facilitators and barriers that were considered
	• methods through which information regarding the facilitators and barriers to implementing recommendations were sought (e.g., feedback from key stakeholders, pilot testing of guidelines before widespread implementation)
	• information/description of the facilitators and barriers that emerged from the inquiry (e.g., certain therapeutic manipulation techniques in TCM guidelines, such as bone-setting techniques, require practitioners to have the appropriate skills to disseminate this recommendation)
	• description of how the information influenced the guideline development process and/or drafting of the recommendations
	Scoring: 7 points will be awarded if all the content listed in “Evaluation” is explained in detail. 2-3 points will be deducted if information related to a criterium under an Item is incomplete. Points will be deducted cumulatively according to how many criteria are not met, and only 1 point will be awarded if no content is described.
19	**The guidelines provide advice and/or tools on how the recommendations can be put into practice.**
	**Description**
	For a set of guidelines to be effective, it needs to be disseminated and implemented with additional materials. For example, specific opinions for implementing the recommendations, such as conditions that need to be tailored to unique implementations, must be provided; Suggestions or practical booklets to guide the specific decoctions and administration methods for Chinese medicinal herbs. Any additional materials must be provided with the guidelines. These may include: a summary document, a quick reference guide, educational tools, results from a pilot test, patient leaflets, or computer support, such as mobile apps or websites.
	**Ratings**
	The item’s content must include the following criteria:
	• an implementation section in the guidelines
	➢ tools and resources to facilitate application
	➢ guideline summary documents
	➢ links to checklists and algorithms
	➢ links to how-to manuals
	➢ solutions linked to barrier analysis (see Item 18)
	➢ tools to capitalize on guideline facilitators (see Item 18)
	• outcomes of pilot tests and lessons learned
	• directions on how users can access tools and resources
	Scoring: 7 points will be awarded if all the content listed in “Evaluation” is explained in detail. 2-3 points will be deducted if information related to a criterium under an Item is incomplete. Points will be deducted cumulatively according to how many criteria are not met, and only 1 point will be awarded if no content is described.
20	**The potential resource implications of applying the recommendations have been considered.**
	**Description**
	The recommendations may require additional resources in order to be applied. For example, there may be a need for more specialized staff, new equipment, or expensive drug treatment. These may have cost implications for health care budgets. The guidelines should contain a discussion regarding recommendations’ potential effects on resources. For example, the TCM guidelines for psoriasis may recommend that the dermatology department implement TCM external therapy or acupuncture for patients. To implement these recommendations, the departments must provide appropriate equipment, and the operators must have the appropriate qualifications.
	**Rating**
	The item’s content must include the following criteria:
	• identification of the types of cost information under consideration (e.g., economic evaluations, drug acquisition costs)
	• methods by which the cost information was sought (e.g., a health economist was part of the guideline development panel, use of health technology assessments for specific drugs, etc.)
	• information/description of the cost information that emerged from the inquiry (e.g., specific drug acquisition costs per treatment course)
	• description of how the information gathered was used to inform the guideline development process and/or drafting of the recommendations
	• Were appropriate experts involved in finding and analyzing the cost information?
	Scoring: 7 points will be awarded if all the content listed in “Evaluation” is explained in detail. 2-3 points will be deducted if information related to a criterium under an Item is incomplete. Points will be deducted cumulatively according to how many criteria are not met, and only 1 point will be awarded if no content is described.
21	**The guidelines present monitoring and/or auditing criteria.**
	**Description:** No changes
	**Rating**
	The item’s content must include the following criteria: No changes
	Scoring: 7 points will be awarded if all the content listed in “Evaluation” is explained in detail. 2-3 points will be deducted if information related to a criterium under an Item is incomplete. Points will be deducted cumulatively according to how many criteria are not met, and only 1 point will be awarded if no content is described.
**Domain 6. Editorial independence**	
22	**The views of the funding body have not influenced the content of the guidelines.**
	**Description:** No changes
	**Rating**
	The item’s content must include the following criteria: No changes
	Scoring: 7 points will be awarded if all the content listed in “Evaluation” is explained in detail. 2-3 points will be deducted if information related to a criterium under an Item is incomplete. Points will be deducted cumulatively according to how many criteria are not met, and only 1 point will be awarded if no content is described.
23	**Competing interests of guideline development group members have been recorded and addressed.**
	**Description:** No changes
	**Rating**
	The item’s content must include the following criteria: No changes
	Scoring: 7 points will be awarded if all the content listed in “Evaluation” is explained in detail. 2-3 points will be deducted if information related to a criterium under an Item is incomplete. Points will be deducted cumulatively according to how many criteria are not met, and only 1 point will be awarded if no content is described.

^a^ No changes: the content did not change from AGREE Ⅱ.

### 3.2 Improving the initial version of AGREE Ⅱ for TCM

Based on the initial version of AGREE Ⅱ for TCM and the literature on TCM clinical guideline development and quality evaluation, the core working group improved the initial version of AGREE Ⅱ for TCM. After discussion, modification was proposed to AGREE Ⅱ Instrument Domain 1 (Items 1, 2), Domain 2 (Items 4, 5), Domain 3 (Items 8, 13), and Domain 5 (Item 20) after discussion (Table 2). Meanwhile, the AGREE Ⅱ Instrument entailed a 7-point rating table, but there were no specific scoring criteria for each point, which could introduce subjectivity, and prolong time of evaluation. The core working group agreed to add details on evaluation rules and deduction criteria in the “Evaluation” part of each item.

**TABLE 2:** The information of the 26 sets of TCM guides included for evaluation.

No.	Name of guidelines	Specialties	Organization	Year of publication
1	Practical guidelines to Chinese medicine preventive treatment of disease on tuina intervention in children with spleen deficiency (formulation)	Chinese medicine preventive treatment·Pediatrics	China association of Chinese Medicine	2017
2	Expert consensus on Chinese medicine diagnosis and treatment of sleep-disordered breathing caused by adenoid hypertrophy in children	Pediatrics	World federation of Chinese medicine societies	2014
3	Evidence-Based Chinese Medicine Clinical Practice Guideline for Stomach Pain in Hong Kong	Gastroenterology	Chinese association of integrative medicine	2017
4	Expert consensus on Chinese medicine diagnosis and treatment of adverse drug reactions of opioids	Oncology	China Anti-Cancer Association	2019
5	Standard for clinical diagnosis and treatment of traditional Chinese medicine for multiple sclerosis/ neuromyelitis optica	Neurology	Beijing provincial association of Chinese Medicine	2018
6	Expert consensus on Chinese medicine diagnosis and treatment of high fever (sepsis)	Emergency	Emergency cooperation group of national administration of traditional Chinese medicine	2014
7	Expert consensus on Chinese medicine diagnosis and treatment of chronic prostatitis	Urology	China association of Chinese Medicine	2015
8	Expert consensus on traditional Chinese medicine diagnosis and treatment of chronic heart failure	Cardiology	Chinese medicine clinical research alliance for coronary heart disease	2014
9	Evidence-Based Chinese Medicine Clinical Practice Guidelines in Prediabetes	Endocrinology	National Chinese medicine clinical research alliance for diabetes of Chinese Medicine clinical research base of national administration of traditional Chinese medicine	2017
10	Guidelines for evidence-based clinical practice of Chinese Medicine in diabetic foot ulcers	Surgery	China association of Chinese Medicine	2017
11	Expert consensus on Chinese medicine diagnosis and treatment of stomach pain	Gastroenterology	Cross strait medical and health exchange association	2016
12	Expert consensus on Chinese medicine diagnosis and treatment of knee osteoarthritis	Orthopedics	China research and promotion of traditional Chinese Medicine	2015
13	Expert consensus on rehabilitation of Chinese medicine for COVID-19	Rehabilitation	World federation of Chinese medicine societies	2020
14	Expert consensus on hierarchical diagnosis and treatment of Chinese medicine for primary osteoporosis in Zhejiang Province	Orthopedics	Zhejiang provincial association of integrative medicine	2018
15	Diagnostic criteria of Chinese medicine syndromes of bronchial asthma (2016 edition)	Respiratory medicine	China association of Chinese Medicine	2016
16	Chinese Medicine rehabilitation Clinical Practice Guidelines for Stroke	Rehabilitation	Chinese medicine rehabilitation standard research base	2019
17	Expert consensus on Chinese Medicine treatment of incomplete abortion	Gynecology	Chinese medical association	2019
18	Chinese Medicine Clinical Practice Guidelines for chronic pelvic inflammatory disease	Gynecology	China academy of Chinese medical sciences and WHO Western Pacific Cooperation Project	2011
19	Chinese Medicine Clinical Practice Guidelines for Hypertension	Cardiology	China academy of Chinese medical sciences and WHO Western Pacific Cooperation Project	2011
20	Expert consensus on the evolution of Chinese medicine syndromes and its concurrent syndromes of chronic obstructive pulmonary disease based on the theory of lung-qi deficiency classification	Respiratory	Anhui provincial association of Chinese Medicine	2015
21	Diagnosis and treatment guidelines of Psoriasis vulgaris in integrative medicine	Dermatology	Guangdong Bureau of Quality and Technical Supervision	2014
22	Chinese medicine guidelines for diagnosis and treatment of Parkinson's disease (tremor and spasm disease)	Neurology	Guangdong provincial association of Chinese Medicine	2021
23	Guidelines for traditional Chinese medicine clinical diagnosis of Novel Coronavirus Pneumonia (COVID-19).	Respiratory medicine	Zhejiang provincial association of integrative medicine	2022
24	Expert consensus on the prevention and treatment for primary osteoporosis with traditional Chinese medicine	Orthopedics	Chinese Medicine and Bone Disease Discipline Group, Osteoporosis Committee, Chinese Gerontological Society	2018
25	Syndrome Differentiation of Diabetes by the Traditional Chinese Medicine according to Evidence-Based Medicine and Expert Consensus Opinion	Endocrinology	Chinese association of integrative medicine	2014
26	Traditional Chinese medicine clinical guidelines for the diagnosis and treatment of mental diseases-Tic disorder	Psychiatry	China association of Chinese Medicine	2018

### 3.3 Expert consensus for the revised version

#### 3.3.1 The experts

The expert group’s primary responsibility was to reach a consensus on the items. Sixteen members were invited into the expert group. They were professionals in TCM or evidence-based medicine from around the world (Beijing, Guangzhou, Shenzhen, Lanzhou, Canada, *etc.*), and are representative in terms of expertise regionally. Among them, 11 were male (68.8%), and five female experts (31.3%). Average age was 32.9 ± 5.21 years (range: 27–46 years), and average length of time invested in related professional fields was 7.5 ± 4.47 years (range: 2–20 years).

#### 3.3.2 Experts’ positivity coefficient and authority

We collected 16 questionnaires, with a responding rate of 100%. None of the questionnaires were invalid, and the experts’ positivity coefficient was 100%.

Generally, experts’ authority coefficient is no less than 0.70, which is acceptable (Li 2001). All experts in this study had authority coefficients no less than 0.838, indicating that they had strong authority on the investigation content.

#### 3.3.3 Concentration of experts’ opinions

The indicators of experts’ opinion concentration include arithmetic mean, full score ratio and grade sum. The meanings of the three indicators are as follows: arithmetic mean is the mean of the evaluation index scores. A mean score of an item is less than or equal to three indicates that this item is unreasonable, and should be revised according to the expert’s opinion before reaching another round of consensus. Full score ratio (Ki) refers to the percentage of experts who “strongly agree” with an item. Ki ≤ 30% indicates that experts deem that the item offers little contribution to the greater evaluation system, and it is one of the conditions for adjusting the items’ content. When an item’s Ki = 0, the item should be deleted. The grade sum (Si) represents the total expert recognition score for an item; it represents the item’s necessity to the greater evaluation system.

The results of the first round of expert surveys show that experts are in strong agreement for all items. The arithmetic mean of all items is greater than or equal to 4.38, the Ki is greater than or equal to 62.5%, and the Si is greater than or equal to 70 (Table 3).

**TABLE 3:** Results of the degree of concentration and coordination of expert opinions.

Evaluation items	N	Mean	Ki(%)	Si	CVi
1. The overall objective(s) of the guidelines are described in detail	16	4.50	62.5	72	0.16
2. The health question(s) covered by the guidelines are described in detail	16	4.38	62.5	70	0.22
3. The population (patients, the general public, etc.) to whom the guidelines are meant to apply is described in detail	16	4.88	87.5	78	0.07
4. The guideline development group includes individuals from all relevant professional groups	16	4.88	87.5	78	0.07
5. Views and preferences of the target population (patients, the general public, etc.) have been sought	16	4.56	62.5	73	0.14
6. The guidelines’ target users are clearly defined	16	4.50	68.8	72	0.20
7. Systematic methods were used to search for evidence	16	4.69	75	75	0.13
8. The criteria for selecting the evidence are clear	16	4.75	81.3	76	0.12
9. The strengths and limitations of the body of evidence are clear	16	4.50	62.5	72	0.16
10. The methods for formulating the recommendations are clear	16	4.81	81.3	77	0.08
11. The health benefits, side effects, and risks have been considered in formulating the recommendations	16	4.69	75	75	0.13
12. There is an explicit link between the recommendations and the supporting evidence	16	4.50	62.5	72	0.16
13. The guidelines were externally reviewed by experts prior to their publication	16	4.81	81.3	77	0.08
14. A procedure for updating the guidelines is provided	16	4.75	75	76	0.09
15. The recommendations are specific and unambiguous	16	4.69	81.3	75	0.17
16. The options for managing the condition or health issue are clearly presented	16	4.69	81.3	75	0.15
17. Key recommendations are easily identifiable	16	4.81	81.3	77	0.08
18. The guidelines describe facilitators and barriers to their application	16	4.75	75	76	0.09
19. The guidelines provide advice and/or tools on how the recommendations can be put into practice	16	4.69	68.8	75	0.1
20. The potential resource implications of applying the recommendations have been considered	16	4.56	62.5	73	0.14
21. The guidelines present monitoring and/or auditing criteria	16	4.56	62.5	73	0.14
22. The views of the funding body have not influenced the guidelines’ content	16	4.94	93.8	79	0.05
23. Guideline development group members’ competing interests have been declared and addressed	16	4.94	93.8	79	0.05

#### 3.3.4 Degree of coordination in experts’ opinions

##### 3.3.4.1 Coefficient of variation

This shows the experts’ fluctuation degree (or coordination degree) for the relative importance of an item. The smaller the CVI, the more coordinated the experts’ evaluation of an item’s importance. The results showed that each item’s Cvi fluctuated between 0.05 and 0.22, the Cvi was small, and the consistency was high (Table 3).

##### 3.3.4.2 Coefficient of coordination (kendall coefficient)

The coefficient of coordination indicates the overall consistency of all experts’ opinions on the importance of all items. We tested the Kendall coordination coefficient, and determined that the coordination coefficient was 0.10, *p* < 0.05. We considered this coordination coefficient statistically significant, with expert consistency and reliable results (Table 3).

### 3.4 The final version of AGREE Ⅱ for TCM

After the first round of investigation, each item in this evaluation system received strong approval from the experts, and the experts’ degree of authority was high. Therefore, a second-round investigation was unnecessary. The experts proposed modifying AGREE Ⅱ Instrument Domain 2 (Items 6), Domain 4 (Items 16) and Domain 5 (Items 19) (Supplementary File S4). Some experts offered suggestions on the expression of some of the items. After being deliberated by the core working group, the expert opinions were adopted to improve the item without affecting the items’ core contents. Finally, under the framework of AGREE II, we finalized the AGREE Ⅱ for TCM.

This new version of AGREE Ⅱ tailored for TCM incorporates the structure, scoring method and main content of the items from AGREE II. The evaluation instrument includes six domains (Scope and Purpose, Stakeholder Involvement, Rigor of Development, Presentation Clarity, Applicability and Editorial Independence), with 23 items in total, and two overall assessment items. We modified and supplied content for the description and rating of several items from AGREE II. This included adjustments and expansion to Items 1, 4, 5, 7, 8, 10, 11, 12, 15, and 19 so that the instrument would be more suitable for clinical TCM guidelines. When providing evaluation examples for each item, we tried to list as many examples from TCM CPGs as possible in items 1, 2, 6, 13, 15, 16, 18, and 20. A total of 16 items were included. We list the full content of all adjusted and expanded items in Supplementary File S4. Any content which has been added to or expanded from AGREE II is underlined in each item. In the “rating” section, we provided detailed explanations on how to score, specifying elements corresponding to each score. A score of one should be given when the information that is relevant to the item is poorly or not reported, or if the authors state explicitly that criteria were not met. A score of seven should be given where all scoring criteria have been met. Two to three points will be deducted if information related to a criterium under an Item is incomplete. Points will be deducted cumulatively according to how many criteria are not met.

## 4 Discussion

In this study, we develop the AGREE Ⅱfor TCM instrument to cover the methodological quality assessment of TCM CPGs based on their characteristics. Currently, adaptation and extension studies of AGREE II have been conducted in several fields, including AGREE -S for surgical interventions (Logullo et al., 2022) and AGREE -China for the evaluation of Chinese CPGs (Wang et al., 2018), *etc.* No extension studies of AGREE Ⅱ for TCM are available yet.

The AGREE Ⅱfor TCM instrument was developed in an accurate and transparent manner in accordance with international standard methods. Methods of guideline evaluation, literature analysis and expert consensus were used during the development of AGREE Ⅱ for TCM. We conducted a systematic search of TCM clinical guidelines, which were classified according to their publishing organizations. Then, we analyzed any characteristics which were different from modern medical guidelines by combining them with each item of AGREE II. Finally, we proposed which contents should be included as items in the new evaluation system. This ensured that the adjusted evaluation system would reflect TCM characteristics as much as possible, and highlighted TCM guidelines’ unique contents and development methods. Distinguishing this part of the study from other TCM guideline quality evaluation studies (Yao, 2016) is that the purpose of conducting TCM guideline evaluation in this study is not to evaluate the quality of TCM guidelines. Rather, it is to understand these items’ applicability to TCM guidelines through evaluation.

By searching the literature on the development and evaluation of TCM guidelines, we collected all problems reported in the literature. After several discussions, we integrated them, and improved the initial version. Based on literature integration and group discussion, we improved them according to the expert opinions and reached an expert consensus. This is how we produced the final version of AGREE Ⅱ for TCM. The research methods above can be used to catalog opinions on TCM guidelines, from content reports to development methodology, and to attain a high degree of expert consensus. This ensures that the research results are scientific, comprehensive and applicable.

During the adjustment and expansion of AGREE II, we added the requirements for, and examples of, TCM guidelines in the description and rating section of some items of the AGREE II instrument to develop the AGREE II for TCM instrument. For example, we used Item one to evaluate whether the purpose of the guidelines was clear, as it was proposed that “the treatment purpose and advantages of the guideline should be described in detail. If the disease in the guidelines is based on Western medicine, its corresponding TCM name needs to be reported. If the disease in the guidelines is based in TCM, its corresponding category of diseases in Western medicine must be reported too”. In terms of highlighting the advantages of TCM, we point out the advantages of TCM in the treatment of a disease must proposed, such as preventing disease recurrence or reducing the side effects of Western medicine. In the context of the widespread use of modern medicine, it is beneficial to explain the therapeutic advantages of TCM in the TCM guidelines to clarify the timing and promote the use of TCM. Item four requires that the development team include experts of TCM or integrated Chinese and Western medicine. Development staff specialization is one of the most important measures for ensuring TCM guidelines’ professionalism, and ensuring that the ideas and methods of guideline development are more compatible to TCM; Item seven requires that systematic methods be applied to retrieve evidence, and information resources include ancient Chinese medicine literature databases such as Chinese medical dictionary and modern Chinese electronic literature databases such as CNKI, Wanfang, VIP and CBM. The keywords must include the TCM disease name corresponding to the disease, such as “traditional Chinese medicine, Chinese patent medicine, herbal medicine “, *etc.* This item is used to evaluate the rationality and comprehensiveness of the retrieval of TCM diagnosis and treatment guidelines. This is done by evaluating whether the retrieval database and keywords have TCM elements; Item 12 requires that there be a clear relationship between the recommendations and supporting evidence, and that there be a clear corresponding relationship between the etiology and pathogenesis, syndrome differentiation and treatment principles, treatment formulas or Chinese patent medicine in the TCM guidelines. The composition of the treatment formulas in the recommendation must have the same name and composition as those in the supporting evidence. The corresponding relationship between TCM theory, principle, method, prescription and medicine must also be considered when formulating recommendations.

AGREE Ⅱ for TCM does not add and delete any item from AGREE II. Rather, only some content related to TCM is added in the “Description” and “how to rate” section, describing specific scoring criteria for evaluation and improving the instruments’ operability. It not only retains the scientificity and rigor of AGREE II, but also highlights the characteristics of TCM, and supplements and improves AGREE II. Applying this evaluation system to the evaluation of TCM CPGs can accurately and scientifically reflect the quality of TCM guidelines, while promoting the normative formulation and clinical application of TCM CPGs. We are planning a validation study of this instrument to see how it works with people outside of this research project. We will promote and disseminate it to more TCM guideline development organizations and hope to continue to modify and improve the AGREE Ⅱfor TCM instrument in the future.

## Data Availability

The original contributions presented in the study are included in the article/Supplementary Material, further inquiries can be directed to the corresponding author.
