# Supplementary material for: AGREE II for TCM: Tailored to evaluate methodological quality of TCM clinical practice guidelines
Source: Front Pharmacol. 2023 Jan 12;13:1057920. doi: 10.3389/fphar.2022.1057920 (PMC9877221; doi:10.3389/fphar.2022.1057920)
Supplement: Supplementary file 1 [file Table1.DOCX]

Distribution of TCM guidelines in different organizations and the distribution of the evaluated guidelines in different organizations

| **Guideline development organization** | **Number of guidelines(set)** | **Number of guidelines for evaluation(set)** |
| --- | --- | --- |
| China academy of Chinese medical sciences and WHO Western Pacific Cooperation Project | 28 | 2 |
| World Federation of Chinese Medicine Societies | 34 | 2 |
| China association of Chinese Medicine | 465 | 5 |
| Chinese association of integrative medicine | 18 | 2 |
| China research and promotion of traditional Chinese Medicine | 2 | 1 |
| Guangdong provincial association of Chinese Medicine | 4 | 1 |
| Guangdong administration for market regulation | 1 | 1 |
| Anhui provincial association of Chinese Medicine | 1 | 1 |
| Beijing provincial association of Chinese Medicine | 3 | 1 |
| Chinese medicine clinical research alliance for coronary heart disease | 1 | 1 |
| Emergency cooperation group of national administration of traditional Chinese medicine | 1 | 1 |
| National Chinese medicine clinical research alliance for diabetes of Chinese Medicine clinical research base of national administration of traditional Chinese medicine | 1 | 1 |
| Cross strait medical and health exchange association | 1 | 1 |
| Jiaxing Standard Quality Construction Promotion Association | 1 | 1 |
| China anti-cancer association | 1 | 1 |
| Zhejiang provincial association of integrative medicine | 1 | 1 |
| Gerontological Society of China | 1 | 1 |
| Chinese medical association | 2 | 1 |
| Chinese medicine rehabilitation standard research base | 1 | 1 |
