# Supplementary material for: AGREE II for TCM: Tailored to evaluate methodological quality of TCM clinical practice guidelines
Source: Front Pharmacol. 2023 Jan 12;13:1057920. doi: 10.3389/fphar.2022.1057920 (PMC9877221; doi:10.3389/fphar.2022.1057920)
Supplement: Supplementary file 2 [file Table2.docx]

The information of the 26 sets of TCM guides included for evaluation

| **No.** | **Name of guidelines** | **specialties** | **Organization** | **Year of publication** |
| --- | --- | --- | --- | --- |
|  | Practical guidelines to Chinese medicine preventive treatment of disease on tuina intervention in children with spleen deficiency (formulation) | Chinese medicine preventive treatment·Pediatrics | China association of Chinese Medicine | 2017 |
|  | Expert consensus on Chinese medicine diagnosis and treatment of sleep-disordered breathing caused by adenoid hypertrophy in children | Pediatrics | World federation of Chinese medicine societies | 2014 |
|  | Evidence-Based Chinese Medicine Clinical Practice Guideline for Stomach Pain in Hong Kong | Gastroenterology | Chinese association of integrative medicine | 2017 |
|  | Expert consensus on Chinese medicine diagnosis and treatment of adverse drug reactions of opioids | Oncology | China Anti-Cancer Association | 2019 |
|  | Standard for clinical diagnosis and treatment of traditional Chinese medicine for multiple sclerosis/ neuromyelitis optica | Neurology | Beijing provincial association of Chinese Medicine | 2018 |
|  | Expert consensus on Chinese medicine diagnosis and treatment of high fever (sepsis) | Emergency | Emergency cooperation group of national administration of traditional Chinese medicine | 2014 |
|  | Expert consensus on Chinese medicine diagnosis and treatment of chronic prostatitis | Urology | China association of Chinese Medicine | 2015 |
|  | Expert consensus on traditional Chinese medicine diagnosis and treatment of chronic heart failure | Cardiology | Chinese medicine clinical research alliance for coronary heart disease | 2014 |
|  | Evidence-Based Chinese Medicine Clinical Practice Guidelines in Prediabetes | Endocrinology | National Chinese medicine clinical research alliance for diabetes of Chinese Medicine clinical research base of national administration of traditional Chinese medicine | 2017 |
|  | Guidelines for evidence-based clinical practice of Chinese Medicine in diabetic foot ulcers | Surgery | China association of Chinese Medicine | 2017 |
|  | Expert consensus on Chinese medicine diagnosis and treatment of stomach pain | Gastroenterology | Cross strait medical and health exchange association | 2016 |
|  | Expert consensus on Chinese medicine diagnosis and treatment of knee osteoarthritis | Orthopedics | China research and promotion of traditional Chinese Medicine | 2015 |
|  | Expert consensus on rehabilitation of Chinese medicine for COVID-19 | Rehabilitation | World federation of Chinese medicine societies | 2020 |
|  | Expert consensus on hierarchical diagnosis and treatment of Chinese medicine for primary osteoporosis in Zhejiang Province | Orthopedics | Zhejiang provincial association of integrative medicine | 2018 |
|  | Diagnostic criteria of Chinese medicine syndromes of bronchial asthma (2016 edition) | Respiratory medicine | China association of Chinese Medicine | 2016 |
|  | Chinese Medicine rehabilitation Clinical Practice Guidelines for Stroke | Rehabilitation | Chinese medicine rehabilitation standard research base | 2019 |
|  | Expert consensus on Chinese Medicine treatment of incomplete abortion | Gynecology | Chinese medical association | 2019 |
|  | Chinese Medicine Clinical Practice Guidelines for chronic pelvic inflammatory disease | Gynecology | China academy of Chinese medical sciences and WHO Western Pacific Cooperation Project | 2011 |
|  | Chinese Medicine Clinical Practice Guidelines for Hypertension | Cardiology | China academy of Chinese medical sciences and WHO Western Pacific Cooperation Project | 2011 |
|  | Expert consensus on the evolution of Chinese medicine syndromes and its concurrent syndromes of chronic obstructive pulmonary disease based on the theory of lung-qi deficiency classification | Respiratory | Anhui provincial association of Chinese Medicine | 2015 |
|  | Diagnosis and treatment guidelines of Psoriasis vulgaris in integrative medicine | Dermatology | Guangdong Bureau of Quality  and Technical Supervision | 2014 |
|  | Chinese medicine guidelines for diagnosis and treatment of Parkinson's disease (tremor and spasm disease) | Neurology | Guangdong provincial association of Chinese Medicine | 2021 |
|  | Guidelines for traditional Chinese medicine clinical diagnosis of Novel Coronavirus Pneumonia (COVID-19). | Respiratory medicine | Zhejiang provincial association of integrative medicine | 2022 |
|  | Expert consensus on the prevention and treatment for primary osteoporosis with traditional Chinese medicine | Orthopedics | Chinese Medicine and Bone Disease Discipline Group, Osteoporosis Committee, Chinese Gerontological Society | 2018 |
|  | Syndrome Differentiation of Diabetes by the Traditional Chinese Medicine according to Evidence-Based Medicine and Expert Consensus Opinion | Endocrinology | Chinese association of integrative medicine | 2014 |
|  | Traditional Chinese medicine clinical guidelines for the diagnosis and treatment of mental diseases-Tic disorder | Psychiatry | China association of Chinese Medicine | 2018 |
