# Supplementary material for: AGREE II for TCM: Tailored to evaluate methodological quality of TCM clinical practice guidelines
Source: Front Pharmacol. 2023 Jan 12;13:1057920. doi: 10.3389/fphar.2022.1057920 (PMC9877221; doi:10.3389/fphar.2022.1057920)
Supplement: Supplementary file 3 [file Table3.DOCX]

**Composition of the expert group**

| **No.** | **Name** | **specialty** | **title** |
| --- | --- | --- | --- |
|  | Geng Li | Clinical Research Methodology | Assistant Researcher |
|  | Hao Luo | Patient Representatives | / |
|  | Hui Li | TCM Neurology | Chief physician |
|  | Huimin Wang | TCM Neurology | Resident doctor |
|  | Jingwen Deng | TCM Dermatology | Attending physician |
|  | Jianxiong Cai | Clinical Research Methodology | Assistant Researcher |
|  | Liang Yao | Evidence-Based Medicine | Assistant Researcher |
|  | Lihong Yang | Evidence-Based Medicine | Assistant Researcher |
|  | Qi Wang | Evidence-Based Medicine | Research Intern |
|  | Runsheng Xie | Standardization of Chinese Medicine | Assistant Researcher |
|  | Sha Yao | Chinese Medicine Bibliography | Resident doctor |
|  | Shaonan Liu | Evidence-Based Medicine | Associate Research Fellow |
|  | Wenjia Chen | Standardization of Chinese Medicine | Research Intern |
|  | Wenjie Xu | TCM Dermatology | Associate Research Fellow |
|  | Yangyang Wang | Standardization of Chinese Medicine | Assistant researcher |
|  | Yun Xia | Chinese Medicine Bibliography | Resident doctor |
