# Supplementary material for: AGREE II for TCM: Tailored to evaluate methodological quality of TCM clinical practice guidelines
Source: Front Pharmacol. 2023 Jan 12;13:1057920. doi: 10.3389/fphar.2022.1057920 (PMC9877221; doi:10.3389/fphar.2022.1057920)
Supplement: Supplementary file 4 [file Table4.docx]

The final version of AGREE Ⅱ^[[1]](#endnote-1)^ for TCM Instrument

| **Item** | **Descriptions and scoring criteria** |
| --- | --- |
| **Domain 1. Scope and purpose** | |
| 1 | **The overall objective(s) of the guidelines are clarified.**  **Description:**  This addresses the potential health effects of a set of guidelines on individuals, patient populations, and society as a whole. The overall objective(s) of the guidelines must be described in detail. For example, guideline contents include disease diagnosis, TCM syndrome diagnosis, treatment and prevention. The expected health benefits from the guidelines must be specific to the clinical problem or health topic. If it is a set of TCM guidelines for a certain disease, the purpose and advantages of TCM treatment must be clarified; the guidelines for using the disease name in Western medicine must specify the corresponding TCM disease name, and the guidelines for using the traditional Chinese medicine disease name must also specify the corresponding disease scope in Western medicine, For example, specific statements would be:   - These guidelines are applicable to patients with psoriasis vulgaris, which is known by names such as psoriasis vulgaris, tinea pinealis, dry tinea and snake lice. - These guidelines are applicable to cough, and common in Western medicine, such as in colds, acute bronchitis, chronic bronchitis, cough variant asthma and postnasal drip syndrome. - In treating this disease, TCM has a clear curative effect, can reduce skin lesions from erythema, scales and itching symptoms, reduce the disease recurrence, and delay the spread of the disease to other parts of the body.   **Rating:**  Item content includes the following criteria:   - health intent(s) (i.e., prevention, screening, diagnosis, treatment, prevention, etc.) - The disease names in both traditional Chinese medicine and Western medicine must be clarified in the guidelines (if appropriate). - advantages, expected benefits or outcomes of TCM treatment - target(s) (e.g., patient population, society)   Scoring:7 points will be awarded if all the content listed in “Evaluation” is explained in detail. 2-3 points will be deducted if information related to a criterium under an Item is incomplete. Points will be deducted cumulatively according to how many criteria are not met, and only 1 point will be awarded if no content is described. |
| 2 | **The health question(s) covered by the guidelines are clarified.**  **Description:**  A detailed description of the health questions covered by the guidelines must be provided, particularly for the key recommendations (see Item 17). For example, specific statements would be:   - Can Chinese native medicine wash-outside in psoriasis treatment reduce the disease’s frequency? - Can biological agents combined with Chinese herbal decoction flakes improve the curative effect in plaque psoriasis treatment?   **Rating:**  Clinical problems cause PICO problems. Item content includes the following criteria:   - Target population (P) - intervention(s) or exposure(s) (e.g., Chinese herbal decoction flakes, Chinese patent medicine or Chinese medicine injection) (I) - comparisons (if appropriate) (C) - outcome(s) (Important patient outcome indicators should reflect the advantages of TCM treatment) (O) - healthcare setting or context   Scoring: 7 points will be awarded if all the content listed in “Evaluation” is explained in detail. 2-3 points will be deducted if information related to a criterium under an Item is incomplete. Points will be deducted cumulatively according to how many criteria are not met, and only 1 point will be awarded if no content is described. |
| 3 | **The population (patients, the general public, etc.) to whom the guidelines are meant to apply is described.**  **Description:** No change^a^  **Rating:**  The item’s content must include the following criteria: No change  Scoring:7 7 points will be awarded if all the content listed in “Evaluation” is explained in detail. 2-3 points will be deducted if information related to a criterium under an Item is incomplete. Points will be deducted cumulatively according to how many criteria are not met, and only 1 point will be awarded if no content is described. |
| **Domain 2. Stakeholder involvement** | |
| 4 | **The guideline development group includes individuals from all relevant professional groups.**  **Description:**  This item refers to the professionals who were involved at some stage of the development process. This may include members of the steering committee, the research team involved in selecting and reviewing/rating the evidence, and individuals involved in formulating the final recommendations. This item excludes individuals who have externally reviewed the guidelines (see Item 13). It also excludes target population representation (see Item 5). Information about the guideline development group’s composition, discipline, and relevant expertise also must be provided.  **Rating:**  The item’s content must include the following criteria:  1) For each member of the guideline development group, the following information is included:   - name - discipline/content expertise (e.g., neurosurgeon, methodologist) - institution (e.g., St. Peter’s Hospital) - geographical location (e.g., Seattle, WA) - a description of the member’s role in the guideline development group   2) Are the members an appropriate match for the topic and scope? The members of the guideline development group must include experts of TCM or integrated traditional Chinese and Western medicine and experts of Chinese herbs. Other potential candidates include relevant clinicians and nurses, content experts, researchers, policy makers, clinical administrators.  3) Is there at least one methodology expert included in the development group (e.g., systematic review expert, epidemiologist, statistician, library scientist, etc.)?  Scoring: 7 points will be awarded if all the content listed in “Evaluation” is explained in detail. 2-3 points will be deducted if information related to a criterium under an Item is incomplete. Points will be deducted cumulatively according to how many criteria are not met, and only 1 point will be awarded if no content is described. |
| 5 | **The views and preferences of the target population (patients, the general public, etc.) have been sought.**  **Description:**  Information about the target population’s healthcare experiences and expectations should inform guideline development. There are various methods for ensuring that these perspectives inform the different stages of guideline development for stakeholders. Examples would include formal consultations with patients/the general public to determine priority topics, these stakeholders’ participation in the guideline development group, or external review by these stakeholders on draft documents. Alternatively, information could be obtained from interviews of these stakeholders, or from literature reviews of patient/general public’s values, preferences or experiences. There must be evidence that some process has taken place, and that stakeholders’ views have been considered.  **Rating:**  Item content must include the following criteria:   - The target population for collecting information must have experience with TCM diagnosis. - statement of strategy used to capture patients’/the general public’s views and preferences (e.g., participation in the guideline development group, literature review of values and preferences, or questionnaire survey) - outcomes/information gathered from patient/public information - description of how the information gathered informed the guideline development process, and/or drafting the recommendations   Scoring: 7 points will be awarded if all the content listed in “Evaluation” is explained in detail. 2-3 points will be deducted if information related to a criterium under an Item is incomplete. Points will be deducted cumulatively according to how many criteria are not met, and only 1 point will be awarded if no content is described. |
| 6 | **The guidelines’ target users are clearly defined.**  **Description:**  The target users must be clearly defined in the guidelines, so that the reader can immediately determine if the guidelines are relevant to them. For example, *TCM Guidelines for Diagnosis and Treatment of Common Diseases in Internal Medicine – Headaches* is applicable to the clinical diagnosis and treatment of headaches in TCM. It is applicable to all levels of TCM (Integrated Traditional Chinese and Western Medicine) medical institutions, as well as medical institutions offering TCM services. These guidelines’ target users may include Chinese medicine practitioners (Integrated Traditional Chinese and Western Medicine) and licensed assistant Chinese medicine practitioners (with the exception of Pediatrics). Clinical practitioners can also refer to them.  **Rating:**  The item’s content must include the following criteria:   - a clear description of the intended guideline audience (e.g. specialists, family physicians, patients, clinical or institutional leaders/administrators) - a description of how the guidelines may be used by their target audience (e.g., to inform clinical decisions, to inform policy, to inform standards of care)   Scoring: 7 points will be awarded if all the content listed in “Evaluation” is explained in detail. 2-3 points will be deducted if information related to a criterium under an Item is incomplete. Points will be deducted cumulatively according to how many criteria are not met, and only 1 point will be awarded if no content is described. |
| **Domain 3. Rigor of development** | |
| 7 | **Systematic methods were used to search for evidence.**  **Description**  Details of the strategy used to search for evidence must be provided; including the search terms used, sources consulted, and dates of the literature covered. Sources may include the tracing of ancient Chinese medical literature records and the searching for modern medical literature. Sources include ancient Chinese medical literature electronic databases (e.g., Chinese Medical Dictionary), Chinese electronic scholarly databases (e.g., CMKI, Wanfang, Wipu, CBM), English electronic scholarly databases (e.g., MEDLINE, ENBASE, CINAHL), other sources including systematic review databases (e.g., Cochrane Library, DARE), manually searched journals, conference proceedings, and other guidelines (e.g., the US National Guideline Clearinghouse, the German Guidelines Clearinghouse). The search terms must include the Chinese Medicine disease name and search terms such as "TCM, Chinese medicine, proprietary Chinese medicine, herbal medicine". The search strategy should be as comprehensive as possible and executed in a manner free from potential biases and detailed enough to be replicable.  **Rating**  The item’s content must include the following criteria:   - named electronic database(s) or evidence source(s) where the search was performed, ancient Chinese medical literature electronic databases (e.g., Chinese Medical Dictionary), Chinese electronic databases (e.g., CMKI, Wanfang, Wipu, CBM), English electronic databases (e.g., MEDLINE, ENBASE, CINAHL). - time periods searched (e.g., January 1, 2004 to March 31, 2008) - search terms used (e.g., text words, indexing terms, sub-headings) - full search strategy (e.g., possibly located in the appendix)   Scoring: 7 points will be awarded if all the content listed in “Evaluation” is explained in detail. 2-3 points will be deducted if information related to a criterium under an Item is incomplete. Points will be deducted cumulatively according to how many criteria are not met, and only 1 point will be awarded if no content is described. |
| 8 | **The criteria for selecting the evidence are clarified.**  **Description**  Criteria for the inclusion and exclusion of evidence at the time of search must be provided. These criteria and the reasons for exclusion/inclusion of evidence must be clarified. For example, the authors of a set of guidelines may decide to include only evidence from randomized controlled trials, and exclude non-Chinese literature.  **Rating**  The item’s content must include the following criteria:   - description of the inclusion criteria, including: - target population (patient, the general public, etc.) characteristics - study design - comparisons (if relevant) - outcomes - language (if relevant) - context (if relevant) - description of the exclusion criteria (if relevant; e.g., Chinese-language-only listed in the inclusion criteria statement could logically preclude non-Chinese-language from being listed in the exclusion criteria statement)   Scoring: 7 points will be awarded if all the content listed in “Evaluation” is explained in detail. 2-3 points will be deducted if information related to a criterium under an Item is incomplete. Points will be deducted cumulatively according to how many criteria are not met, and only 1 point will be awarded if no content is described. |
| 9 | **The strengths and limitations of the body of evidence are clarified.**  **Description:** No change  **Rating:** No change  Scoring: 7 points will be awarded if all the content listed in “Evaluation” is explained in detail. 2-3 points will be deducted if information related to a criterium under an Item is incomplete. Points will be deducted cumulatively according to how many criteria are not met, and only 1 point will be awarded if no content is described. |
| 10 | **The methods for formulating the recommendations are clarified.**  **Description**  A description of the methods used to formulate the recommendations and how final decisions were reached should be provided. For example, methods may include a voting system, informal consensus, and formal consensus techniques (e.g., Delphi, Glaser techniques). Experts participating in the drafting of recommendations must include experts of TCM, integrated traditional Chinese and Western medicine. Any areas of disagreement and the methods of resolving them should be specified. The TCM diagnosis must have clear sources and basis, such as expert consensus or literature.  **Rating**  The item’s content must include the following criteria:   - description of the recommendation development process (e.g., background information on the experts who participate in drafting the recommendations; the members of the expert group must include experts in TCM or integrative Chinese and Western medicine; steps used in the modified Delphi technique; voting procedures that were considered) - outcomes of the recommendation development process (e.g., the extent to which consensus was reached using the modified Delphi technique, outcome of voting procedures) - description of how the process influenced the recommendations (e.g., how the results of the Delphi technique influenced final recommendation, alignment with recommendations and the final vote)   Scoring: 7 points will be awarded if all the content listed in “Evaluation” is explained in detail. 2-3 points will be deducted if information related to a criterium under an Item is incomplete. Points will be deducted cumulatively according to how many criteria are not met, and only 1 point will be awarded if no content is described. |
| 11 | **The health benefits, side effects, and risks have been considered in formulating the recommendations.**  **Description**  The guidelines should consider health benefits, side effects, and risks when formulating the recommendations.  For example, a set of guidelines on breast cancer management may include a discussion on the overall effects on various final outcomes. These may include: survival, quality of life, adverse effects, and symptom management, or a discussion comparing one treatment option to another. There should be evidence that these issues have been addressed. The characteristics and advantages of traditional Chinese medicine treatment must be explained; the health benefits and deficiencies of any Chinese medicinal herb with toxic side effects must be analyzed.  **Rating**  The item’s content must include the following criteria:   - supporting data and report of benefits - the characteristics and advantages of traditional Chinese medicine treatment - supporting data and report of harm/side effects/risks - reporting of the balance/trade-off between benefits and harm/side effects/risks, analysis of the health benefits and deficiencies of any Chinese medicinal herb with toxic side effects. - recommendations reflecting considerations of both benefits and harm/side effects/risks   Scoring: 7 points will be awarded if all the content listed in “Evaluation” is explained in detail. 2-3 points will be deducted if information related to a criterium under an Item is incomplete. Points will be deducted cumulatively according to how many criteria are not met, and only 1 point will be awarded if no content is described. |
| 12 | **There is an explicit link between the recommendations and the supporting evidence.**  **Description**  An explicit link between the recommendations and the evidence on which they are based should be included in the guidelines. The user of the guidelines should be able to identify the components of the body of evidence relevant to each recommendation. There must be clear correspondence between the etiology and pathogenesis, syndrome differentiation and treatment principles, therapeutic formulas or proprietary Chinese medicine in TCM guidelines. The composition of the therapeutic formulas in the recommendations must have the same name and composition as those in the evidence.  **Ratings**  The item’s content must include the following criteria:   - the guidelines describe how the guideline development group linked and used the evidence to inform recommendations (When evidence is lacking or a recommendation is informed primarily by consensus of opinion by the guideline group, rather than the evidence, is this clearly stated and described?) - each recommendation is linked to a key evidence description/paragraph and/or reference list - recommendations linked to evidence summaries, evidence tables in the results sections of guidelines; the etiology and pathogenesis, syndrome differentiation and classification in the TCM guidelines have clear correspondence with treatment principles, treatment prescriptions or proprietary Chinese medicines - the composition of the therapeutic formula in the recommendation must have the same name and composition as the formula in the supporting evidence.   Scoring: 7 points will be awarded if all the content listed in “Evaluation” is explained in detail. 2-3 points will be deducted if information related to a criterium under an Item is incomplete. Points will be deducted cumulatively according to how many criteria are not met, and only 1 point will be awarded if no content is described. |
| 13 | **The guidelines have been externally reviewed by experts prior to their publication.**  **Description**  Guidelines should be reviewed externally before they are published. Reviewers should not have been involved in the guideline development group. Reviewers should include experts in clinical TCM, as well as methodological experts. Target population representatives (e.g., patients, the general public) may also be included. A description of the methodology used to conduct the external review should be presented, which may include a list of the reviewers and their affiliations.  **Rating**  The item’s content must include the following criteria:   - a clear description of the intended guideline audience’s (e.g., specialists, family physicians, patients, clinical or institutional leaders/administrators) purpose and intent regarding the external review (e.g., to improve quality, gather feedback on draft recommendations, assess applicability and feasibility, disseminate evidence) - methods utilized for the external review (e.g., rating scale, open-ended questions) - description of the external reviewers (e.g., number, type of reviewers, affiliations) - outcomes/information gathered from the external review (e.g., summary of key findings) - description of how the information gathered was used to inform the guideline development process and/or drafting of recommendations (e.g., the guideline panel considered the review results in drafting the final recommendations)   Scoring: 7 points will be awarded if all the content listed in “Evaluation” is explained in detail. 2-3 points will be deducted if information related to a criterium under an Item is incomplete. Points will be deducted cumulatively according to how many criteria are not met, and only 1 point will be awarded if no content is described. |
| 14 | **A procedure for updating the guidelines is provided.**  **Description:** No change  **Rating**  The item’s content must include the following criteria: No changes  Scoring: 7 points will be awarded if all the content listed in “Evaluation” is explained in detail. 2-3 points will be deducted if information related to a criterium under an Item is incomplete. Points will be deducted cumulatively according to how many criteria are not met, and only 1 point will be awarded if no content is described. |
| **Domain 4. Clarity of presentation** | |
| 15 | **The recommendations are specific and unambiguous.**  **Description**  A recommendation must provide a concrete and precise description of which option is appropriate in which situation and in what population group, as informed by the body of evidence. The usage and dosage of the Chinese medicinal herb must be specified in the recommendation; the diagnostic criteria of TCM syndromes must be clear; there must be indications and detailed administration methods for TCM therapies; Chinese herbal medicines must have clear sources if they are non-self-formulated; there must be explanations for the special TCM terms that affect the guidelines.  It is important to note that in some instances, evidence is not always clear-cut and there may be uncertainty about the best care option(s). In this case, the uncertainty should be stated in the guidelines.  **Rating:**  The item’s content must include the following criteria:   - statement of the recommended action - identification of the intent or purpose of the recommended action (e.g., to improve quality of life, to mitigate side effects) - the content of the recommendations is clear and unambiguous - identification of the relevant population (e.g., patients, the general public) - caveats or qualifying statements, if relevant (e.g., patients or conditions for whom the recommendations would not apply)   Scoring: 7 points will be awarded if all the content listed in “Evaluation” is explained in detail. 2-3 points will be deducted if information related to a criterium under an Item is incomplete. Points will be deducted cumulatively according to how many criteria are not met, and only 1 point will be awarded if no content is described. |
| 16 | **The options for management of the condition or health issue**  **are clearly presented**  **Description**  A set of guidelines that targets the management of a disease must consider all possible options for screening, prevention, diagnosis or treatment of the condition it covers. These possible options must be clearly presented in the guidelines.  For example, a recommendation on the management of depression may contain the following treatment alternatives:  a. Chinese medicine treatment  b. Acupuncture  c. Acupoint application  **Rating**  The item’s content must include the following criteria:   - a clear description of the intended guideline audience (e.g. specialists, family physicians, patients, clinical or institutional leaders/administrators), a description of options - description of the population or clinical situation most appropriate for each option   Scoring: 7 points will be awarded if all the content listed in “Evaluation” is explained in detail. 2-3 points will be deducted if information related to a criterium under an Item is incomplete. Points will be deducted cumulatively according to how many criteria are not met, and only 1 point will be awarded if no content is described. |
| 17 | **Key recommendations are easily identifiable**  **Description:** No changes  **Rating**  The item’s content must include the following criteria: No changes  Scoring: 7 points will be awarded if all the content listed in “Evaluation” is explained in detail. 2-3 points will be deducted if information related to a criterium under an Item is incomplete. Points will be deducted cumulatively according to how many criteria are not met, and only 1 point will be awarded if no content is described. |
| **Domain 5. Applicability** | |
| 18 | **The guidelines describe facilitators and barriers to their application.**  **Description**  There may be existing facilitators and barriers that will influence the application of guideline recommendations. For example:  i. A set of guidelines on stroke may require that care be coordinated through stroke units and stroke services. There may be a special funding mechanism in the region to enable the creation of stroke units.  ii. A set of guidelines on diabetes in primary care may require that patients are seen and followed up in  diabetic clinics. There may be an insufficient number of clinicians available in a region to enable the establishment of clinics.  **Rating**  The item’s content must include the following criteria:   - identification of the facilitators and barriers that were considered - methods through which information regarding the facilitators and barriers to implementing recommendations were sought (e.g., feedback from key stakeholders, pilot testing of guidelines before widespread implementation) - information/description of the facilitators and barriers that emerged from the inquiry (e.g., certain therapeutic manipulation techniques in TCM guidelines, such as bone-setting techniques, require practitioners to have the appropriate skills to disseminate this recommendation) - description of how the information influenced the guideline development process and/or drafting of the recommendations   Scoring: 7 points will be awarded if all the content listed in “Evaluation” is explained in detail. 2-3 points will be deducted if information related to a criterium under an Item is incomplete. Points will be deducted cumulatively according to how many criteria are not met, and only 1 point will be awarded if no content is described. |
| 19 | **The guidelines provide advice and/or tools on how the recommendations can be put into practice.**  **Description**  For a set of guidelines to be effective, it needs to be disseminated and implemented with additional materials. For example, specific opinions for implementing the recommendations, such as conditions that need to be tailored to unique implementations, must be provided; Suggestions or practical booklets to guide the specific decoctions and administration methods for Chinese medicinal herbs. Any additional materials must be provided with the guidelines. These may include: a summary document, a quick reference guide, educational tools, results from a pilot test, patient leaflets, or computer support, such as mobile apps or websites.  **Ratings**  The item’s content must include the following criteria:   - an implementation section in the guidelines - tools and resources to facilitate application - guideline summary documents - links to checklists and algorithms - links to how-to manuals - solutions linked to barrier analysis (see Item 18) - tools to capitalize on guideline facilitators (see Item 18) - outcomes of pilot tests and lessons learned - directions on how users can access tools and resources   Scoring: 7 points will be awarded if all the content listed in “Evaluation” is explained in detail. 2-3 points will be deducted if information related to a criterium under an Item is incomplete. Points will be deducted cumulatively according to how many criteria are not met, and only 1 point will be awarded if no content is described. |
| 20 | **The potential resource implications of applying the recommendations have been considered.**  **Description**  The recommendations may require additional resources in order to be applied. For example, there may be a need for more specialized staff, new equipment, or expensive drug treatment. These may have cost implications for health care budgets. The guidelines should contain a discussion regarding recommendations’ potential effects on resources. For example, the TCM guidelines for psoriasis may recommend that the dermatology department implement TCM external therapy or acupuncture for patients. To implement these recommendations, the departments must provide appropriate equipment, and the operators must have the appropriate qualifications.  **Rating**  The item’s content must include the following criteria:   - identification of the types of cost information under consideration (e.g., economic evaluations, drug acquisition costs) - methods by which the cost information was sought (e.g., a health economist was part of the guideline development panel, use of health technology assessments for specific drugs, etc.) - information/description of the cost information that emerged from the inquiry (e.g., specific drug acquisition costs per treatment course) - description of how the information gathered was used to inform the guideline development process and/or drafting of the recommendations - Were appropriate experts involved in finding and analyzing the cost information?   Scoring: 7 points will be awarded if all the content listed in “Evaluation” is explained in detail. 2-3 points will be deducted if information related to a criterium under an Item is incomplete. Points will be deducted cumulatively according to how many criteria are not met, and only 1 point will be awarded if no content is described. |
| 21 | **The guidelines present monitoring and/or auditing criteria.**  **Description:** No changes  **Rating**  The item’s content must include the following criteria: No changes  Scoring: 7 points will be awarded if all the content listed in “Evaluation” is explained in detail. 2-3 points will be deducted if information related to a criterium under an Item is incomplete. Points will be deducted cumulatively according to how many criteria are not met, and only 1 point will be awarded if no content is described. |
| **Domain 6. Editorial independence** | |
| 22 | **The views of the funding body have not influenced the content of the guidelines.**  **Description:** No changes  **Rating**  The item’s content must include the following criteria: No changes  Scoring: 7 points will be awarded if all the content listed in “Evaluation” is explained in detail. 2-3 points will be deducted if information related to a criterium under an Item is incomplete. Points will be deducted cumulatively according to how many criteria are not met, and only 1 point will be awarded if no content is described. |
| 23 | **Competing interests of guideline development group members have been recorded and addressed.**  **Description:** No changes  **Rating**  The item’s content must include the following criteria: No changes  Scoring: 7 points will be awarded if all the content listed in “Evaluation” is explained in detail. 2-3 points will be deducted if information related to a criterium under an Item is incomplete. Points will be deducted cumulatively according to how many criteria are not met, and only 1 point will be awarded if no content is described. |

^a^No changes: the content did not change from AGREE Ⅱ.

1. The AGREE Next Steps Consortium.(2009). Appraisal of guidelines for research & evaluation II. Hamilton: The AGREE Research Trust [↑](#endnote-ref-1)
